# Understanding Self‐Leadership in Nursing: A Descriptive Qualitative Study

**DOI:** 10.1111/jan.70684

**Published:** 2026-07-17

**Authors:** Katja Pursio, Pauline van Dorssen‐Boog, Anu Nurmeksela, Emilia Raula, Tarja Kvist

**Affiliations:** ^1^ Department of Nursing Science, Faculty of Health Sciences University of Eastern Finland Kuopio Finland; ^2^ Research Centre for Management of Employability Zuyd University of Applied Sciences Sittard the Netherlands; ^3^ Wellbeing Services County of Central Finland Jyväskylä Finland

**Keywords:** autonomous actions, focus groups, nurses, nursing practice, qualitative study, self‐leadership, thematic framework

## Abstract

**Aim:**

To describe nurses' perceptions and to deepen the conceptual understanding of nurses' self‐leadership in nursing.

**Design:**

A qualitative descriptive interview study.

**Methods:**

Data were collected between December 2024 and February 2025 in one Finnish Wellbeing Services County through six online focus group interviews with a total of 17 nurses from diverse care settings. The interviews followed a structured thematic framework. The abductive analysis began with a deductive coding frame, which was iteratively refined through inductive interpretation. The resulting dimensions were further quantified by examining their frequency of occurrence.

**Results:**

All ten dimensions outlined in the theoretical framework were evident in the nurses' narratives of self‐leadership. In addition, two new main dimensions were identified. Across nurses' accounts of self‐leadership, the most frequently recurring dimensions were autonomous actions, collaboration, professional capability, time management, and balancing the workload.

**Conclusion:**

Although theoretical self‐leadership strategies can be identified in nursing practice, they do not fully capture the everyday, context‐bound realities of the profession. In practice, nurses enact self‐leadership through concrete, situationally embedded behaviours.

**Implications for the Profession:**

The normative orientation of self‐leadership theory prescribes how individuals should regulate themselves to achieve optimal performance. However, prioritizing these prescribed strategies may narrow the analytical lens and obscure alternative conceptualizations of self‐leadership. This study deepens the understanding of self‐leadership in nursing by identifying its key dimensions as nurses themselves describe them and by contributing a new perspective to the existing theoretical framework. The findings emphasize that nurses' self‐leadership is largely grounded in situationally embedded behaviours, which underscores the practical relevance of self‐leadership for everyday nursing practice and its potential to support high‐quality care.

**Reporting Method:**

The study was reported in accordance with the Standards for Reporting Qualitative Research.

**Patient or Public Contribution:**

No patient or public involvement.

## Abstract (highlights)

**What Is Already Known:**

- The three most widely recognized self‐leadership strategies are behaviour‐focused strategies, natural reward strategies, and constructive thought pattern strategies. In addition to these strategies, the literature has introduced dimensions that expand self‐leadership capacity and higher‐level forms of self‐leadership.
- Using self‐leadership strategies can contribute to positive outcomes in relation to performance, innovative work behaviour, and well‐being.

**What This Paper Adds:**

- The study identifies dimensions of self‐leadership as described by the nurses themselves, thereby deepening our understanding of the concept within nursing and contributing a new perspective to the existing theoretical framework of self‐leadership.
- The findings demonstrate that nurses' self‐leadership is primarily centred on concrete and situationally embedded behaviours such as autonomous actions, collaboration, professional capability, effective time management, and workload balancing.

## 1 Introduction

In the past decades, professional autonomy has been highlighted in nursing research as an important resource for the motivation and performance of nurses (Pursio et al. 2021; Qamar et al. 2025). Hospitals and policymakers are searching for ways to empower nurses structurally. However, improving professionals' autonomy is challenging because nursing involves numerous laws, regulations, and guidelines that govern practice and is also fundamentally characterized by interprofessional collaboration (ICN 2021). Nevertheless, employees have many opportunities to manage their motivation and behaviour by employing cognitive and behavioural self‐regulation strategies and to make autonomous choices (Manz 2015; van Dorssen‐Boog et al. 2020). To do so, employees need to effectively act upon the given responsibilities, which requires self‐leadership competences (van Dorssen‐Boog et al. 2020; Pursio et al. 2025).

Self‐leadership can be defined as an individual's capacity to regulate their own thoughts and behaviour to enhance their performance. It is strongly based on autonomous reflections and actions for which specific behavioural, cognitive, and motivational self‐influencing strategies are assumed to be helpful (Manz 2015). Behavioural strategies such as self‐observation can help to bring focus to behaviour, and cognitive strategies such as positive self‐talk can help increase self‐efficacy for improved performance. Natural reward strategies, which refer to both behavioural and thought strategies, aim to make activities more intrinsically motivating. A growing body of evidence shows that the use of these self‐leadership strategies can contribute to performance and innovative work behaviour, which is partly explained by increased self‐efficacy, job satisfaction, and work engagement (Knotts and Houghton 2021). For nursing, some evidence shows that self‐leadership can contribute to positive outcomes in relation to performance and well‐being. However, relevant research is relatively scarce (Pursio et al. 2025).

The normative character of self‐leadership theory has the advantage of prescribing how one should ideally lead oneself to attain optimal performance. However, focusing research on these specific strategies can cause one to overlook other perspectives on self‐leadership, which can easily lead to the misinterpretation of study results (van Dorssen 2022). In line with this, Manz (2015) proposed that self‐leadership theory could be expanded with other types of strategies, for instance, emotional self‐leadership, collaboration, and adopting a healthy lifestyle. Moreover, specific job types may require specific types of self‐influencing strategies to enable good performance. For instance, in clinical practice, collaboration can be an important strategy because the goal is the shared benefit of the patient, to which different professionals contribute their expertise (Wei et al. 2022).

This research aims to gain knowledge of and deeper insight into how nurses describe self‐leadership to develop our understanding of the concept within this profession. This understanding will broaden the perspective on self‐leadership among nurses, as it will draw from both prescriptive and descriptive approaches to self‐leadership (van Dorssen 2022).

## 2 Theoretical Framework

Early definitions of self‐leadership emphasized self‐management and the mechanisms of self‐direction as a substitute for external leadership. Manz (1986) proposes that employees should be able to manage their own job tasks while being less dependent on directions from a leader. Over time, the concept has expanded to include the behavioural, motivational, and cognitive strategies that guide one toward optimal functioning and well‐being. These strategies help make activities more intrinsically motivating so that the activities align with individual goals and interests. Moreover, self‐leadership theory recognizes that there will always be job tasks and situations that are not intrinsically motivating although they will still need to be completed. For these cases, one can use self‐management strategies to regulate behaviour and realize goals (Manz 2015; Neck et al. 2019). Unlike mere self‐management, self‐leadership is grounded in positive psychology, focusing on the conditions and processes that foster flourishing and optimal functioning while supporting overall well‐being (Goldsby et al. 2021). Typically, three main strategies are identified: behaviour‐focused strategies, natural reward strategies, and constructive thought pattern strategies. In addition to these strategies, the literature has introduced dimensions that expand self‐leadership capacity and higher‐level forms of self‐leadership (Manz 2015; Goldsby et al. 2020; van Dorssen‐Boog et al. 2020). The theoretical framework of this study is described below and summarized in Figure 1.

**FIGURE 1:**
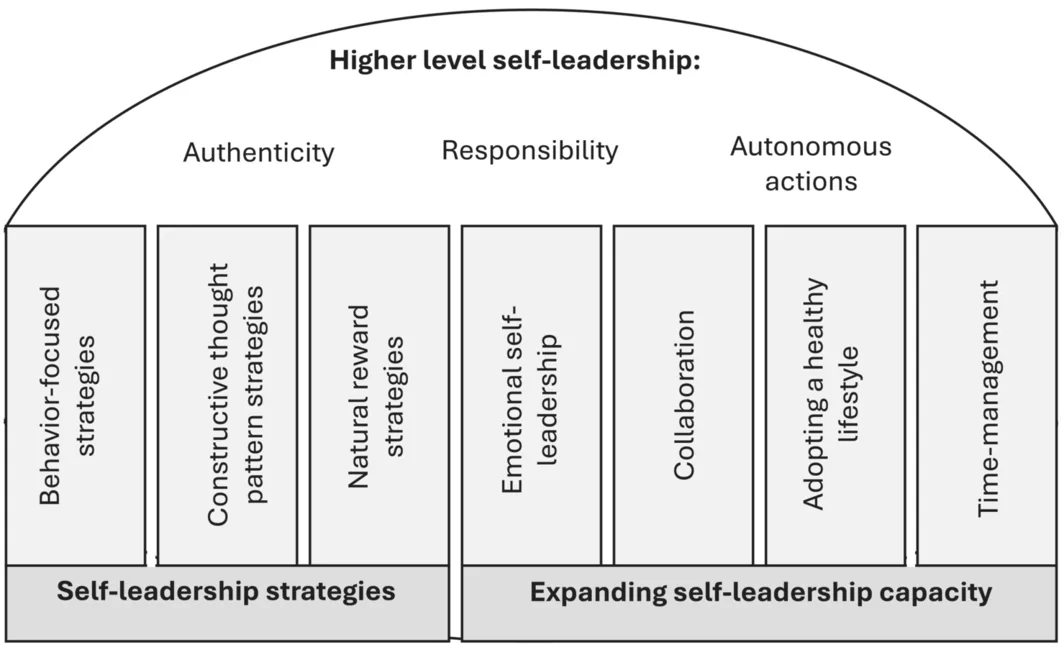
Theoretical framework of the study.

*Behaviour‐Focused Strategies* help direct and regulate individual behaviour (Manz 2015). These strategies promote disciplined action both for tasks that one must do to optimize performance and for tasks that one willingly undertakes to enhance vitality based on self‐determined goals (van Dorssen‐Boog et al. 2021). *Self‐observation, self‐goal setting, self‐reward, self‐punishment, and self‐cueing* are identified in the literature as the primary concrete actions associated with this strategy and managing one's behaviour (Neck et al. 2019). Self‐observation involves monitoring one's own behaviour to increase self‐awareness and support personal goal setting. Strategies such as self‐reward, self‐punishment, and self‐cueing help guide behaviour toward desired outcomes through the use of different approaches (Manz 2015). Self‐rewards may be tangible or internal, while self‐punishment reflects self‐directed criticism aimed at improving future behaviour. Self‐cues focus on creating reminders and reducing environmental distractions that can impede progress toward one's goals (Neck et al. 2019).

*Constructive thought pattern strategies* focus on shaping mental activity to promote a more positive and supportive mindset toward one's actions and situations. These strategies aim to reframe cognitive processes to enhance optimism and, in turn, improve individual performance. A key element involves identifying dysfunctional beliefs and assumptions and replacing them with more constructive alternatives (Manz 2015). These strategies manifest through *self‐talk*, *evaluating beliefs and assumptions*, and *visualizing successful performance*. Self‐talk refers to what we say to ourselves out loud or in our minds to encourage ourselves. Becoming more aware of these inner dialogues helps individuals reduce negative or irrational thoughts and replace them with more constructive ones. Mental imagery involves picturing successful performance beforehand, which has been shown to increase the likelihood of achieving desired outcomes. Strengthening positive beliefs includes recognizing and challenging harmful thinking patterns, such as all‐or‐nothing thinking or discounting one's achievements, and replacing them with more adaptive interpretations (Neck et al. 2019).

*Natural Reward Strategies*, also referred to as intrinsic motivational strategies, are an energizing and positive source of optimizing performance (Manz 2015). These strategies involve not only recognizing and valuing the positively reinforcing aspects of one's work but also creating a better job for oneself. Individuals can actively increase natural rewards in their daily activities by performing tasks in ways that align more closely with their preferences, strengths, and sources of intrinsic enjoyment (Neck et al. 2019). They emphasize aspects of work that are personally enjoyable and meaningful, thereby guiding behaviour through intrinsic motivation and the natural rewarding qualities of the tasks. Furthermore, they promote motivating experiences of competence and self‐control (Manz 2015). Natural reward strategies refer to the intentional structuring of one's environment to include objects, activities, and social interactions that promote the enactment of desirable behaviours (van Dorssen‐Boog et al. 2020).

### 2.1 Expanding Self‐Leadership Capacity

Until now, self‐leadership research has mainly focused on investigating the use of behaviour‐focused strategies, constructive thought strategies, and natural reward strategies, which are often measured using the well‐established Revised Self‐Leadership Questionnaire (Houghton and Neck 2002). However, scholars have identified the potential importance of other domains of strategies such as emotional self‐leadership, collaboration, adopting a healthy lifestyle, and time management.

*Emotional Self‐Leadership* refers to an individual's capacity to regulate their own emotions and to shape emotional experiences (Manz et al. 2016). Emotional self‐leadership strategies are crucial during demanding emotional situations as they help manage emotions in ways that uphold professionalism and support the reframing of emotionally charged situations (Manz et al. 2016; Goldsby et al. 2020). Emotion regulation strategies can be diverse. They include avoidance of stimuli or cognitive change strategies, which involve altering thoughts to influence emotional response. Mindfulness helps regulate emotions by allowing one to be present in the moment and accept situations as they are, without judgement. Mindfulness‐based self‐leadership strengthens emotional regulation by integrating cognitive and behavioural strategies such as self‐observation and the evaluation of thoughts (Sampl et al. 2017).

*Collaboration* can serve as an intentional self‐leadership strategy that strengthens individual effectiveness. By purposefully engaging in joint work with others, individuals gain access to a broader pool of expertise, social support, and collaborative synergies that facilitate goal attainment (Neck et al. 2019). Moreover, collaboration enables individuals to compensate for personal limitations and extend their capabilities beyond the confines of their own knowledge and skills (Manz 2015).

*Adopting a Healthy Lifestyle* can be used as a self‐leadership strategy to enhance personal effectiveness. Attending to one's physical and psychological well‐being strengthens the capacity to perform work tasks efficiently and sustainably (Neck et al. 2019). Regular physical activity and a nutritious diet help maintain work ability and improve resilience to stress (Manz 2015). Moreover, a health‐promoting lifestyle has been shown to facilitate energy balance, recovery, and self‐efficacy (Bjerke 2024).

Effective *time management* entails understanding the time required for tasks, maintaining a realistic awareness of one's personal time‐related resources, and making purposeful choices that manifest in practices such as prioritizing and focusing on the right tasks. Managing time pressure is important for successful decision‐making. Under time pressure, individuals can regulate their own cognition and behaviour through self‐leadership (Goldsby et al. 2020).

### 2.2 Higher‐Level Self‐Leadership

Complementing self‐leadership strategies, the theoretical framework incorporates higher‐level domains, with authenticity and responsibility being the most significant domains (Manz 2015). Higher‐level self‐leadership is embedded within all strategies; however, that occurs with the specific reflection on the *what* and *why* of activities to make sure that one can fully endorse one's own decisions and behaviour. Higher‐level self‐leadership reflects the autonomous functioning of the individual (van Dorssen‐Boog et al. 2020).

*Authenticity* refers to having a clear sense of self and engaging in behaviours that reflect one's true nature and align with deeply held personal values. Authentic individuals focus on higher‐level goals that clarify the *why* behind actions, allowing them to go beyond mere ‘should‐do’ behaviour rooted in formal job responsibilities or external directives. This *why* perspective broadens the focus of self‐leadership by incorporating deeper personal factors such as values, ethics, and well‐being, and it requires understanding what one genuinely values and believes in, thus allowing one to make choices consistent with that understanding (Manz 2015). Authentic actions are based on autonomous choices and are therefore those actions that individuals recognize as their own and for which they are willing to take personal responsibility (Ryan and Deci 2017). It is further strengthened when individuals act in ways that utilize their personal strengths (van Dorssen‐Boog et al. 2021).

*Responsibility* encompasses objectives directed toward the broader common good, including consideration for employees, stakeholders, and society at large. These virtues can be cultivated through self‐leadership strategies. Enhancing responsibility within the framework of self‐leadership requires critical reflection on one's personal values (Manz 2015) as well as on ethical decision‐making and behaviour (Goldsby et al. 2021). As with authenticity, responsibility involves clarifying the *why* behind one's actions and deepening the understanding of self‐leadership in relation to professionalism and organizational goals (Manz 2015).

*Autonomous Actions* reflect the actual behaviour of individuals who function on the basis of self‐leadership (van Dorssen‐Boog et al. 2020). Autonomous actions denote genuine autonomous functioning, which is reflected in taking responsibility for one's tasks, solving work‐related problems in a self‐responsible and independent way, and managing routine job‐related challenges (van Dorssen‐Boog et al. 2020; Pursio et al. 2021).

## 3 The Study

This study aims to describe nurses' perceptions and to deepen the conceptual understanding of nurses' self‐leadership in nursing. The following specific research question is addressed:

How do nurses describe self‐leadership in professional practice?

## 4 Methods

### 4.1 Study Design

A qualitative descriptive interview study was undertaken to obtain an in‐depth understanding of nurses' experiences of self‐leadership within their professional practice. The study employed an integrated analytical approach that combined deductive and exploratory elements, allowing for the simultaneous assessment of theoretically informed assumptions and the identification of novel, data‐driven dimensions. This dual strategy enhanced the study's explanatory depth and supported a more comprehensive interpretation of self‐leadership (Proudfoot 2022).

### 4.2 Settings and Participants

The study was conducted in a Finnish Wellbeing Services County that provides social, healthcare, and rescue services for approximately 273,000 residents and employs an average of 2150 registered nurses. In Finland, social and healthcare services have been integrated into a single system to ensure coordinated and comprehensive support for people. All registered nurses across primary healthcare and specialized medical care formed the recruitment base for this study, with ‘specialized medical care’ referring to hospital‐based services that medical specialists provided for more serious or complex health problems. Practical nurses, nursing students, managerial staff, and temporary substitute nurses were excluded from the study. Recruitment was conducted through a contact person who distributed the study information and interview invitations to nurse leaders; these were subsequently forwarded to nurse managers and then to registered nurses within their units. Participation was voluntary, and interviews were arranged through direct contact with the researcher. This approach was considered appropriate for the aims of the study, as it enabled the inclusion of participants with relevant professional experience and the willingness to reflect on self‐leadership in nursing in depth, thereby supporting the generation of rich, information‐oriented qualitative data.

### 4.3 Data Collection

Data gathering was conducted between December 2024 and February 2025 through six focus group interviews involving two to four participants. The relatively small group sizes were intentional to facilitate in‐depth discussion and to ensure that each participant had adequate opportunity to share their experiences (Krueger and Casey 2014). In addition, practical considerations, such as participants' availability and work schedules, influenced the formation and sizes of the groups. Seventeen registered nurses from diverse care settings and multiple units participated in the study. The interviews were conducted using a pre‐designed structured thematic framework with a set of supporting questions (Table 1). During the interviews, the nurses were asked about externally imposed expectations as well as perceived obstacles to their self‐leadership. This portion of the data was excluded from the present study, combined with corresponding interview data collected from nurse leaders on expectations and obstacles, analysed as a separate entity using inductive content analysis, and reported in another study. The two studies thus constitute distinct bodies of work without overlap in the use of data across the analyses.

**TABLE 1:** Interview guide.

Interview theme	Supporting questions
Nurses' self‐leadership in practice	How would you describe self‐leadership? What does self‐leadership mean in your work? How does self‐leadership appear in your work? An example? In what situations do your own self‐leadership skills become visible at work? An example? What kinds of skills does self‐leadership require from you?

All interviews addressed the same main topic; however, the order of the supporting questions and the emphases of the responses varied with the focus group (Krueger and Casey 2014). Focus group interviews lasted between 34 and 57 min, resulting in a total of 4 h and 40 min of recorded material, which was transcribed into 86.5 pages (Aptos, font size 12, line spacing 1.0). The first author carried out all the interviews online with the assistance of the fourth author; both authors possessed experience in the nursing context and in conducting a qualitative interview study. The researchers had no prior acquaintance with the interviewees.

### 4.4 Data Analysis

Abductive content analysis was used to analyse data (Elo and Kyngäs 2008). The analysis was conducted manually using Excel spreadsheets, beginning with the extraction of expressions from the data that addressed the research question. The first and fourth authors participated in this process. The nurses' original expressions related to the description and definition of self‐leadership were distilled into short phrases. These simplified expressions formed the codes (*n* = 256). The original data were read multiple times to ensure that the dataset was fully considered in the analysis.

The analysis began with a deductive approach by constructing a structured matrix based on the theoretical framework of self‐leadership. The main dimensions (*n* = 10) formed behaviour‐focused strategies, constructive thought pattern strategies, natural reward strategies, emotional self‐leadership, collaboration, adopting a healthy lifestyle, time management, authenticity, responsibility, and autonomous actions. Subsequently, the codes were classified under these 10 dimensions using theory‐driven insights. A new, as yet unnamed dimension was added to the end of the matrix for codes that described nurses' self‐leadership but did not conceptually fit within the dimensions of the theoretical framework (Elo and Kyngäs 2008). The research team members reviewed and analysed all the codes multiple times to ensure a shared interpretation.

In the next phase, the codes (*n* = 53) that did not conceptually fit the existing dimensions were analysed inductively. Those with similar content were grouped into subdimensions (*n* = 5) and then organized into main dimensions (*n* = 2) to complement the previously identified dimensions of self‐leadership (Elo and Kyngäs 2008). The examples of codes related to the dimensions are presented in Table 2.

**TABLE 2:** Dimensions of nurses' self‐leadership with exemplary codes and quotes from the data.

Dimensions	Examples of the codes
Behavioural‐focused strategies Self‐observation Self‐goal setting Self‐cueing	Evaluating one's own performance Setting goals and giving directions to one's own work Using a task list as a memory aid
Constructive thought pattern strategies	Believing in oneself
Self‐talk	Recognizing that not everything can be handled alone
Evaluation of beliefs and assumptions	Accepting that not everything can be influenced
Natural reward strategies	Working in a workplace that find pleasant Finding suitable working methods for oneself Challenging oneself occasionally
Emotional self‐leadership	Managing emotions so they do not affect work Sharing mental workload with colleagues Tolerating uncertainty
Collaboration	Consulting when necessary Participating in interprofessional problem‐solving Sharing tasks fairly with colleagues
Adopting a healthy lifestyle	Preparing in advance to sustain endurance during the workday Taking care of oneself Considering own resources when planning work shifts (lengths of shift)
Time‐management	Scheduling one's own work Prioritizing work tasks Focusing on essential matters
Authenticity	Using one's own personality in performing tasks Working by utilizing one's own strengths Trusting one's own competence
Responsibility	Acting responsibly Behaving professionally Addressing and raising shortcomings when necessary
Autonomous actions	Controlling one's own work Performing work independently Completing work thoroughly in one go
Balancing the workload	Taking breaks during workday
Microbreaks	Focusing on other aspects of life during free time
Work‐life balance	Leaving work matters at work
Professional capability Information management Comprehensive oversight Self‐improvement	Knowing how to search for information Understanding the whole picture and acting accordingly Maintaining competence and developing oneself

Finally, the number of codes was calculated across all dimensions following the principles of the initial phases of quantification, and presented as basic descriptive statistics (frequencies, percentages), highlighting the prominence of each dimension within the interview data (Banha et al. 2022).

### 4.5 Rigour and Trustworthiness

Credibility, transferability, confirmability, and dependability were considered at multiple stages of the research process to reinforce methodological rigour and uphold the study's trustworthiness (Enworo 2023). A total of 17 registered nurses provided a robust dataset. Each participant enriched the data with their subjective experiences, and reaching data saturation might not necessarily have been possible. However, similarities were observed across the data, and the later interviews were considered to largely repeat contents that had emerged earlier (Kyngäs et al. 2020). The participants were familiar with the content of nursing work as they had the same professional role. Moreover, as they worked in diverse care environments, they were able to describe, discuss, and reflect on the research phenomenon from multiple perspectives. Two researchers were present at each interview to maintain methodological consistency and confirm that the results correctly reflected the participants' perspectives. A structured interview guide (Table 1) was consistently applied across interviews, maintaining methodological dependability. The recordings allowed attention to detail in the discussions, while verbatim transcriptions ensured that the information could be revisited as needed.

An abductive approach allowed the complementing of the deductive analysis with inductive reasoning, ensuring that the entire dataset was examined. The data were revisited iteratively during analysis to ensure that expressions were interpreted within their contexts, thereby reducing interpretive bias (Kyngäs et al. 2020). Definitions of nurses' self‐leadership were first examined deductively against existing theory, and data classification was refined through research group discussions. Expressions falling outside the theoretical framework were reviewed several times within the research group prior to inductive analysis. Credibility and confirmability were strengthened through multidisciplinary expertise, reflexive practices, and consensus‐building discussions, which also helped minimize individual bias. All members of the research team possessed methodological expertise, experience, and theoretical knowledge of the topic, further supporting the rigour of the study. Careful reporting and the presentation of quotations alongside the results enhanced the dependability of the analysis and results.

### 4.6 Ethical Approval and Informed Consent

The Committee on Research Ethics at the University of Eastern Finland granted ethical approval on September 12, 2024 (Statement No. 17/2024), and the Wellbeing Services County granted study permission on October 20, 2024 (Hyvaks, 20.10.2024). The approval covered the use of the data in sub‐studies and across different stages of reporting. Participants received a detailed information sheet in advance outlining the study, their participation, and their rights. Participation was voluntary, and informed consent was confirmed at the beginning of each interview, including permission to use the interview data in related studies addressing different research questions. All procedures adhered to the principles of the Declaration of Helsinki.

## 5 Results

### 5.1 Characteristics of Participants

A total of 17 registered nurses participated in the study. Their ages ranged from 30 to 64 years, with a mean age of 46.9 years. Most participants completed their nursing degree at a university of applied sciences (*n* = 10). Five nurses obtained their qualification at the former college level, two of them additionally completed a specialization degree, and two participants held a master's degree. On average, the respondents had 16.6 years of work experience, with a range from 2 to 39 years.

### 5.2 Deductive Findings

All ten dimensions outlined in the theoretical framework were evident in the nurses' narratives of their self‐leadership. Examples of dimension‐related codes are presented in Table 2.

#### 5.2.1 Behaviour‐Focused Strategies

In terms of behaviour‐focused strategies, the nurses' descriptions of self‐leadership centred primarily on self‐observation. They reported monitoring their own actions and performance, paying attention to deficiencies, and regularly reviewing their goals. Self‐goal setting was also identified as a form of self‐leadership that provided direction for their work. The nurses employed various self‐cues, such as writing down unfinished tasks and using to‐do lists as memory aids during busy days.

I guess I'm the kind of person who always has goals, a certain direction, certain plans and … I've noticed it's the same for me at work too. (RN 16)

The way you manage all that buzzing around during the day—it can mean just getting things out of your head and onto paper, so you're not trying to actively hold everything in mind like ‘this and this, and then I still had to do that and that.’ Instead, it's all written down, and then you just start doing. (RN 4)

#### 5.2.2 Constructive Thought Pattern Strategies

The nurses described cognitive strategies in terms of evaluating their beliefs and assumptions. In their daily work, for instance, they reported processing distressing thoughts, allowing time for reflection, and accepting that not everything can be managed alone or even influenced at all. The nurses also highlighted that they value and believe in themselves, which could be seen as a form of positive self‐talk. Likewise, they understood self‐leadership as embodying courage.

We don't really have the privilege of deciding for ourselves how many hectic days we get in a row. (RN 5)

#### 5.2.3 Natural Reward Strategies

The nurses described that they were able to work in a way that suits them, within given boundaries and in accordance with their own resources. To maintain their well‐being at work, they focused on aspects of the job that bring them joy. Regularly challenging themselves was also mentioned as part of self‐leadership. In addition, they emphasized the importance of working in a unit that they had chosen themselves. They noted that their choice was influenced, for example, by the unit's location, available resources, and a positive work atmosphere.

Of course, a workplace that you're actually interested in, where you feel like it's nice to be … you want to do things even better. (RN 6)

#### 5.2.4 Emotional Self‐Leadership

For the nurses, the regulation of emotions was a key aspect of their practice. It was reflected in emotional intelligence and in the ability to manage one's emotions during the workday, including maintaining inner calm under rush and pressure, and remaining present in the moment. Equally important was the ability to share and process emotions after demanding or stressful situations through discussions with colleagues. Emotional self‐leadership involved tolerating uncertainty, accepting unfinished tasks, and accepting situations that were beyond one's influence. Moreover, emotional self‐leadership was evident in the nurses' ability to accept that they could not know everything, that mistakes could happen, and that they should be compassionate toward themselves.

If there's been a challenging situation or a really busy moment at work, being able to talk it through with colleagues, going over what happened … helps so it doesn't follow you home, or at least not for too long. It's about processing things and getting peer support in that moment. (RN 9)

You have to be able to tolerate uncertainty … you don't know what the work shift will be like when you go in. (RN 4)

#### 5.2.5 Collaboration

The nurses described their work as being based on collaboration, and self‐leadership took place within that context. In collaboration with their colleagues, self‐leadership referred to the ability to share tasks according to what felt most suitable for each person, as well as to divide tasks fairly and collectively and to delegate them when necessary. In addition, asking for and offering help, utilizing others' expertise, and the mutual exchange of feedback were considered important self‐leadership skills.

As a group, we decide how to divide certain tasks, like … what can be done alongside the rest of the work. (RN7)

Self‐leadership also included communicating within an interprofessional team, where nurses needed to take an equal role. Maintaining open discussion and participating in joint problem‐solving and decision‐making were mentioned to require an active role from nurses. Understanding when consultation was required and whom to consult was a highlighted self‐leadership skill. In collaborative work, the emphasis was on being a good team player, the ability to listen, mutual trust, and acceptance of different personalities and ways of working.

You really come across all kinds of situations, in every direction. Sometimes you just have to call here, there, and everywhere to figure out how to move forward. (RN 6)

#### 5.2.6 Adopting a Healthy Lifestyle

Some remarks focused on adopting a healthy lifestyle. The nurses placed emphasis on recognizing their own resources and supporting their personal well‐being through various recovery methods during their free time. Preparing for the workday in ways that promoted endurance was also highlighted. Outdoor activities, sufficient sleep, and healthy nutrition were active choices that nurses could make. In addition, paying attention to mental well‐being and setting appropriate boundaries could help prevent exhaustion.

When I know I've got several days coming up that will be all sitting and answering phone calls, then I, for example, go outside more so I can stay focused the whole day and manage just sitting still. (RN 17)

I've tried different ways. Sometimes I just relax, exercise, study, and so on … recharging is made up of different things, depending on the moment. We need different ways. (RN 10)

#### 5.2.7 Time Management

Time management within nurses' self‐leadership manifested in the ability to prioritize. The nurses described themselves as continuously assessing which tasks required immediate attention and which could be postponed. Moreover, they scheduled their own work, planned an optimal order of tasks, critically assessed their time management, and focused on essential duties aligned with their job description and professional role.

In daily work, self‐leadership kind of starts from the type of patient or patients I'm caring for. What their needs are? And from there I build the structure of my day. (RN 5)

#### 5.2.8 Authenticity

With reference to authenticity, the nurses described self‐leadership as valuing their profession and personal skills, trusting in their own competence, and drawing on their experience. They considered it important to know themselves, their own strengths and weaknesses, and to make use of their personal strengths in nursing practice. Working in a way that drew on one's own personality emerged as an essential aspect of nurses' self‐leadership.

The work is based on self‐leadership; everyone does it in their own style, in their own way, and each person is open to their own strengths. (RN 2)

#### 5.2.9 Responsibility

Responsibility, as an individual word, was repeatedly mentioned in the nurses' definitions of self‐leadership. This dimension was also described with regard to behaving professionally, acting in accordance with ethical principles, focusing on each patient individually, and knowing one's own duties. The nurses also highlighted the responsibility to address shortcomings, report issues to supervisors when necessary, and contribute to developing practices further.

You pay attention to shortcomings and fix them or at least pass on suggestions for improvement. And you act responsibly … in a way that you don't think, ‘I'm just an employee’. (RN 1)

#### 5.2.10 Autonomous Actions

The nurses described self‐leadership both as having autonomy and as engaging in actions related to autonomy. These descriptions particularly emphasized organizing daily work within their tasks and responsibilities, making autonomous decisions regarding patient care and their own job description, and performing nursing practice independently. In this context, self‐directedness was highlighted, along with understanding one's options within their professional role and maintaining control over the overall practice.

Within the rules, I can currently lead and carry out my own work quite freely. We each have our own roles and based on the weekly schedule, how the tasks have been assigned for the week, I can control my own work accordingly. (RN 13)

Independent work planning was considered a central part of a nurse's role. By this, the nurses meant planning their own shifts and organizing an efficient workflow according to patient needs. They were expected to independently initiate and complete tasks in a timely manner. Self‐leadership was also associated with striving to complete tasks efficiently and without unnecessary delay so that unresolved work does not carry over into free time.

In our unit we've got clear guidelines, and within those we basically plan our own shifts. Then we just go ahead and plan and carry out the actual work pretty independently. (RN3)

### 5.3 Inductive Findings

In addition to the theory‐driven findings, two new main dimensions were identified: balancing the workload and professional capability. Balancing the workload comprised two subdimensions, namely, microbreaks and work–life balance, whereas professionalism encompassed information management, comprehensive oversight, and self‐improvement. Figure 2 presents the inductive findings.

**FIGURE 2:**
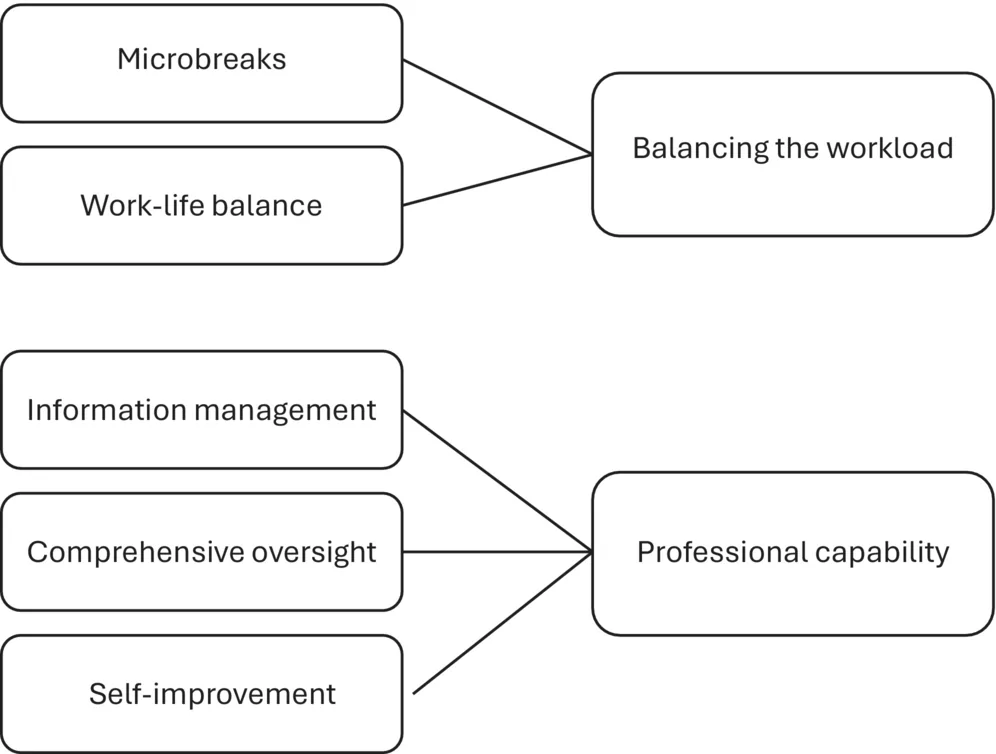
Two inductively analysed dimensions of nurses' self‐leadership and their subdimensions.

#### 5.3.1 Balancing the Workload

The nurses indicated that active self‐leadership included ensuring appropriate breaks during the workday. In a hectic work environment, such pauses were not necessarily provided externally; rather, taking them was each nurse's own responsibility. *Microbreaks* were described as essential for maintaining high‐quality and safe patient care. The nurses also emphasized the importance of declining additional responsibilities when they did not have sufficient time resources.

Taking breaks too, probably. I can kind of pace my workday, structure it in a way that's reasonably suited to me. (RN13)

Similarly, managing one's workload in a manner that allowed for adequate recovery between shifts was considered an important component of self‐leadership. The participants reported the need to intentionally refrain from taking overtime, to leave work matters at the workplace, and to direct their attention to other aspects of life during their free time. Setting boundaries and maintaining *work–life balance* were seen as key elements of self‐leadership.

Work is one part of life, and private life is another … When I shut down my computer and close the door, the work really does stay here. (RN 16)

#### 5.3.2 Professional Capability

*Information management* in nurses' daily practice was characterized as the ability to search for relevant information to respond to patients' needs and resolve complex situations. The nurses described that they used different guidelines and applied professional knowledge in their work. In addition, they employed information technology for documenting patient care.

That you know how to look up information from the right and reliable sources if there's something you don't know. (RN 2)

An aspect of self‐leadership noted by nurses was having *comprehensive oversight* of patient situations, in which they continuously assessed the patient's overall condition and the effectiveness of the care provided. They identified deviations from the expected course and considered appropriate follow‐up actions. Furthermore, they possessed a clear understanding of their job responsibilities and were able to perform them appropriately.

We ourselves assess and carry out the patient's care. And we look at the overall situation, whether there are things that may need to be directed elsewhere. For example, we might ask physiotherapists, nutritionists, and so on to come. (RN 1)

The nurses also described *self‐improvement* as a part of self‐leadership. By this, they referred to identifying gaps in their competence and asking necessary questions. They expressed a desire to maintain and develop their competence and to learn new things so they could advance in their careers. From a self‐leadership perspective, they emphasized their active responsibility in using quieter moments at work for self‐improvement, as well as in planning and allocating time for this purpose.

When I need new tools for my job, I go around asking like, ‘hey, could I maybe join this training,’ because I'd like to strengthen this and that area. I can see there's a real need for it, and it's the kind of thing that keeps my motivation, energy, and skills up. (RN 5)

### 5.4 Frequency of Self‐Leadership Dimensions

In the nurses' accounts of self‐leadership, autonomous actions emerged as the most prominent dimension (*n* = 55, 20.8%), followed by collaboration (*n* = 37, 14.5%), professional capability (*n* = 29, 11.3%), time management (*n* = 25, 9.4%), and balancing the workload (*n* = 24, 9.4%). The nurses described least often self‐leadership through adopting a healthy lifestyle (*n* = 7, 2.7%), constructive thought pattern strategies (*n* = 8, 3.1%), and behaviour‐focused strategies (*n* = 9, 3.5%). The frequency of the self‐leadership dimension in nurses' self‐leadership is illustrated in Figure 3.

**FIGURE 3:**
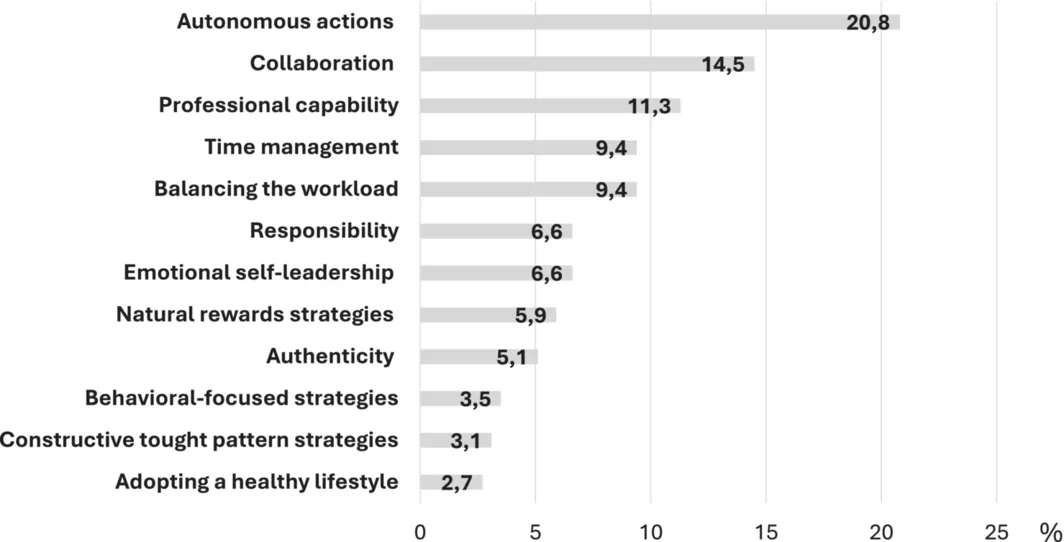
Frequency of the self‐leadership dimensions in nurses' self‐leadership (%).

## 6 Discussion

In the past decades, normative self‐leadership theory focused on specific sets of strategies that could be applied in all kinds of professions to optimize performance. Meanwhile, this study aimed to investigate how nurses described self‐leadership in their professional practice. With the use of a theory‐driven analytical approach, the findings were presented through the established strategies and dimensions of self‐leadership (e.g., Manz 2015; van Dorssen‐Boog et al. 2020) while foregrounding the realities specific to nursing work. All theoretical dimensions were identifiable in the nurses' narratives. However, the three strategies commonly emphasized in previous research; behaviour‐focused strategies, constructive thought patterns, and natural reward strategies (Houghton and Neck 2002), were only marginally reflected as explicit strategies. Instead, the nurses most prominently described autonomous actions that reflected the concrete behaviours required to accomplish work tasks effectively. Collaboration and time management also frequently emerged as indicators of expanded self‐leadership capacity. Additionally, two new dimensions that were not part of the existing framework, balancing the workload and professional capability, were identified.

This study has shown that nurses emphasize aspects of self‐leadership that differ from common conceptualizations in the literature. Interestingly, based on the findings of our study, autonomy appears to be closely intertwined with, and may even be embedded within, multiple dimensions of self‐leadership, in addition to emerging as a distinct construct in its own right. Nursing is characterized by an increasing number of complex tasks, including the need to make rapid decisions with limited resources and under time pressure (Goldsby et al. 2020). Many of the nurses' authentic expressions portray autonomy as a feature of the job itself, specifically the freedom to determine how their work is carried out. However, this does not necessarily imply that nurses routinely perform autonomous actions (van Dorssen 2022). When describing self‐leadership, the nurses emphasize the experience of autonomy rather than its behavioural manifestations. Consequently, self‐leadership in nursing appears both as an inherent characteristic of the role and as an aspect that nurses actively seek to influence at the individual level. Consistent with the view that self‐leadership also reflects actual autonomous functioning (van Dorssen‐Boog et al. 2020), the nurses in this study have further described self‐leadership as organizing their daily work, making independent decisions in the patient's best interest, and shaping their own job roles.

This study also shows that autonomous action and professional responsibility are mirrored in the way nurses manage their time by continuously prioritizing work tasks, scheduling their own work, and being able to focus on essential matters. Goldsby et al. (2020) proposed a model suggesting that self‐leadership and time management, together with psychosocial care, contributed to reducing perceived time pressure. The findings of the present study lend further support to this model, as nurses explicitly characterized time management as a central element of their self‐leadership. In their accounts, they portrayed time management as the capacity to structure and prioritize work tasks within constrained temporal resources. From this perspective, time management can be understood as a self‐leadership strategy that facilitates self‐regulatory processes and enhances the effective use of one's capabilities.

In line with our expectations, nurses strongly highlight collaboration as part of self‐leadership. Patient care in healthcare is fundamentally based on interprofessional cooperation (Wei et al. 2022), and effective teamwork and seamless nurse–physician relations are important to nurses (Zhao et al. 2025). The nurses have predominantly described how they share tasks collectively and utilize others' expertise. They have also described self‐leadership, in relation to collaboration, through mutual trust and participation in joint discussions. While Neck et al. (2019) describe team self‐leadership through the use of self‐leadership strategies, such as shared behavioural strategies (e.g., team self‐observation, team self‐goal setting) and collective cognitive strategies (e.g., team self‐talk, team beliefs, and assumptions), the participants in the present study do not mention those strategies. However, nurses can benefit from these types of strategies if they are aware of them and know how to use them. For instance, van Dorssen (2022) finds in an intervention study that healthcare workers in elderly care and home care can conjointly develop self‐leadership for team purposes such as reducing work‐related stress and improving work engagement.

Behaviour‐focused strategies appear through references to self‐observation, goal setting, and self‐cues. In our interpretation, neither self‐reward nor self‐punishment is mentioned at all. Interestingly, a previous study (Pursio et al. 2026) has shown that self‐punishment, in particular, is relatively common among nurses. However, this earlier finding is based on data collected using a structured instrument (Houghton and Neck 2002), in which nurses evaluate their self‐leadership by using predefined items. It is therefore possible that, when describing self‐leadership in their own words, nurses do not perceive self‐punishment as part of self‐leadership or do not consider it meaningful to emphasize. Thus, it can be inferred that one must first become aware of a specific self‐leadership strategy for them to employ it, thereby underscoring the need for interventions that enhance the awareness of self‐leadership strategies (van Dorssen‐Boog et al. 2021).

Still, the nurses recognize the relevance of natural reward strategies as a way to improve or sustain their intrinsic motivation for nursing. They highlight the importance of working in units that suit their preferences and of seeking joy and positive challenges to maintain vitality. These findings are documented in prior nursing research (van Dorssen 2022; Pursio et al. 2026) and reinforce earlier understanding that natural reward strategies are relevant for optimizing motivation and functioning. In addition, a recent literature review emphasizes that nurses' individual cognition and behaviours, such as self‐efficacy, autonomy, trust in colleagues, use of personal strengths, and clinical leadership behaviours, contribute to job satisfaction. These cognitive and behavioural factors largely mediate the relationship between nurses' job satisfaction and various environmental conditions (Zhao et al. 2025).

While the dimensions identified beyond the theoretical framework in this study are well established in the nursing context and do not constitute novel standalone concepts, they contribute to extending self‐leadership research by incorporating perspectives derived from nurses' clinical practice. In particular, professional capability, as described by nurses, appears to be oriented toward strengthening professionalism in nursing. Professionalism may be viewed as an outcome reflecting nurses' performance, whereas the identified subdimensions (information management, comprehensive oversight, and self‐improvement) can be understood as self‐influencing strategies that support its development. It is important to note that these subdimensions identified in this study represent only a limited part of the broader and multifaceted nature of nurses' professionalism. In previous studies, professionalism is reflected in nurses' attitudes, competencies, and behaviours in clinical practice, grounded in the laws, concepts, and standards that guide care (Kaya and Boz 2019). It also manifests in how nurses perform their work, demonstrate pride in their profession, and contribute to patient safety, quality of care, and positive outcomes (Kurucová et al. 2023). Notably, self‐improvement has consistently emerged as the least realized dimension of professionalism (Kurucová et al. 2023), and the suppression of professional growth has been identified as an aspect that increases intentions to leave the profession (Valizadeh et al. 2018). Previous studies also show a positive association between nurses' autonomy and professionalism (Al‐sbehat et al. 2024), and autonomy itself has even been identified as a central component of nursing professionalism (Pursio et al. 2021). Professionalism is inherently characterized by responsible and ethical conduct, suggesting that it aligns closely with what Manz (2015) describes as ‘higher‐level self‐leadership’. Therefore, within this framework, professional capability can be seen as a dimension of nurses' self‐leadership.

Given that contemporary nursing work is characterized by resource shortages and high workload (World Health Organization 2025), it is understandable that nurses also highlighted strategies such as taking microbreaks and safeguarding one's work–life balance in order to balance their workload. In nursing, the workload can fluctuate on a daily basis, for instance due to the severity of patients' illnesses and the complexity of care that is needed (Alghamdi 2016; Morris et al. 2007). Moreover, recent crises, exceptional circumstances, and the economic uncertainty affecting healthcare have subjected organizational structures and leadership to continuous change, which has led to elevated stress and burnout (Golfenshtein et al. 2026). Balancing the workload could therefore be one of the key self‐leadership dimensions for nurses that sustain their well‐being and general functioning. Efforts to support nurses' well‐being have been limited by an overemphasis on individual resilience and a lack of tools for proactive fatigue detection, systematic monitoring, and organizational support (Fox et al. 2026). As nurse fatigue has become normalized in many contexts, the responsibility for coping and maintaining professional functioning has increasingly shifted to individual nurses. Indeed, the nurses in our study supported Manz's (2015) description of healthy lifestyle behaviours as an integral part of self‐leadership, while also resonating with Bjerke's (2024) view of a healthy lifestyle as a form of self‐leadership that fosters self‐efficacy and supports recovery. Consistently with these perspectives, nurses highlighted the importance of balancing workload through practices such as taking microbreaks during the working day and maintaining work–life balance.

This study addresses an identified knowledge gap and offers new insights by broadening the idealized nature of self‐leadership theory toward a more context‐sensitive understanding of how self‐leadership is enacted in nursing practice. The five most frequently described dimensions of self‐leadership in this study (autonomous actions, collaboration, professional capability, time management, and balancing the workload) are in some respects challenging to interpret because they do not consistently represent strategies but instead function as prerequisites or outcomes of self‐leadership. Nurses often describe these dimensions, for example, in terms of competence or capability. Additionally, the dimensions are interrelated, as can be seen in independent work planning and the organization of an efficient workflow, which are autonomous actions that may also reflect aspects of time management. Distinguishing clearly between authenticity and natural reward strategies also presents conceptual challenges, as they may be interpreted as reflecting the same underlying phenomenon.

Hence, although self‐leadership may be viewed as a normative concept in its current form, excessively broadening it risks introducing conceptual ambiguity. Several of the identified dimensions already constitute established constructs in their own right. Expanding the concept to encompass a wide range of elements related to managing oneself, others, organizations, or work processes may, accordingly, obscure its boundaries rather than clarify them. Expansion may be called for; however, it requires a stronger theoretical and philosophical foundation, as perspectives derived solely from how a professional group describes a concept are not sufficient to define it comprehensively. The findings of this study further emphasize that, in nursing, self‐leadership is described in practical terms and is predominantly understood as the management of one's own work.

### 6.1 Limitations

Certain limitations need to be considered when interpreting these results. The study is limited to registered nurses in a single Wellbeing Services County in Finland. A sample of 17 nurses represents only a small proportion of the profession. The nurses' descriptions of self‐leadership are subjective, reflecting the qualitative approach of the study (Enworo 2023). While this can also be viewed as a strength in relation to the research question, the cultural context and specific characteristics of the Finnish healthcare environment may nevertheless shape the findings. Secondly, online video interviews may limit some participants' ability to articulate their thoughts as fully and equitably as they can in traditional in‐person interviews. In a focus group setting, it is also possible that the participants provide socially desirable responses or refrain from expressing divergent views. The small number of participants in the focus groups may, in turn, have contributed to a certain lack of diversity in the discussions.

Finally, the data contain multiple layers of meaning and nuance, and the analysis of such material inevitably involves some interpretive bias. Moreover, the theoretical approach applied in the analysis may have influenced the interpretations and steered the understanding of authentic expressions toward theory‐confirming readings.

### 6.2 Recommendations for Further Research

A comprehensive concept analysis of self‐leadership in nursing will be necessary in the future. It is essential to define what attributes, antecedents, and consequences are central to nurses' self‐leadership and thereby gain a clearer understanding of the specific strategies nurses employ in their self‐leadership. It is also important to note that professional capability and workload balancing emerged as two inductively derived self‐leadership dimensions in this study and are among the most frequently described in the nurses' narratives. As previously noted, we do not consider these dimensions to be new or unique to nurses; rather, the ways in which they were articulated reflect how nurses typically express and highlight core elements of self‐leadership within nursing practice. Nevertheless, these dimensions should be taken into account when advancing self‐leadership theory in the future, as they warrant further in‐depth investigation and continued empirical examination using appropriate methodological approaches. Moreover, we emphasize that instruments tailored to nursing practice are needed, as self‐leadership manifests in ways that are specific to the nursing context and cannot be adequately captured with generic measures. Therefore, further development and validation of nursing‐specific instruments will be essential going forward.

### 6.3 Implications for Practice

The normative orientation of self‐leadership theory clearly prescribes how individuals should regulate themselves to achieve optimal performance. However, prioritizing these prescribed strategies may narrow the analytical lens and obscure alternative conceptualizations of self‐leadership, thereby increasing the risk of misinterpreting empirical findings. This study deepens the understanding of self‐leadership in nursing by identifying its key dimensions as nurses themselves describe them and by contributing a new perspective to the existing framework. Importantly, the findings emphasize that nurses' self‐leadership is largely grounded in concrete, situationally embedded behaviours, such as autonomous decision‐making, collaboration, professional competence, effective time management, and workload balancing, which underscores the practical relevance of self‐leadership for everyday nursing practice and its potential to support sustainable and high‐quality care.

## 7 Conclusions

The theoretical framework of self‐leadership is applicable to nursing, but it remains inadequate in its current form. Although this study demonstrates that the established theoretical strategies are identifiable in nursing practice, they do not adequately capture the everyday, context‐bound realities of the profession. In practice, nurses enact self‐leadership through concrete and situationally embedded behaviours such as autonomous actions, collaboration, professional capability, effective time management, and workload balancing. Accordingly, the findings of this study suggest that self‐leadership theory may benefit from further development, with increased attention to context‐specific approaches that better capture the complexities of nursing work.

## Author Contributions

**Katja Pursio:** conceptualization and design, methodology, data collection, data analysis, tables and figures, writing – original and revised draft. **Pauline van Dorssen‐Boog:** conceptualization and design, methodology, data analysis, writing – original and revised draft. **Anu Nurmeksela:** conceptualization and design, methodology, data analysis, writing – original and revised draft. **Emilia Raula:** data collection, data analysis, writing – original and revised draft. **Tarja Kvist:** conceptualization and design, methodology, data analysis, writing – original and revised draft. All authors: final approval and agreement to be accountable for all aspects of the work.

## Funding

This study was supported by Työsuojelurahasto (The Finnish Work Environment Fund).

## Ethics Statement

Ethical approval was obtained from the Committee on Research Ethics at the University of Eastern Finland on September 12, 2024 (Statement No 17/2024), while Wellbeing services county provided study permission on October 20, 2024.

## Consent

The authors have nothing to report.

## Conflicts of Interest

The authors declare no conflicts of interest.

## Data Availability

The data is not publicly available due to privacy or ethical restrictions.
